# Supplementary material for: The impact of neighborhood-level racial and economic segregation on low-risk cesarean delivery among Black, White, and Biracial (Black/White) individuals
Source: BMC Pregnancy Childbirth. 2025 Nov 25;26:4. doi: 10.1186/s12884-025-08502-2 (PMC12764071; doi:10.1186/s12884-025-08502-2)
Supplement: Supplementary file 1 — Supplementary Material 1. [file 12884_2025_8502_MOESM1_ESM.docx]

**Supplemental Table of Contents**

**Supplemental Table 1.** Data sources of Study of Outcomes in Mothers and Infants

| **Variable** | **Data source** | **Notes/ICD codes** |
| --- | --- | --- |
| Race/ethnicity | Birth records | Non-Hispanic White, Black, Two or more races: Black and White |
| Mode of delivery | Birth records | 01 = Cesarean – primary  11 = Cesarean – primary, with trial of labor attempted  21 = Cesarean – primary, with vacuum  31 = Cesarean – primary, with vacuum and trial of labor attempted  02 = Cesarean – repeat  12 = Cesarean – repeat, with trial of labor attempted  22 = Cesarean – repeat, with vacuum  32 = Cesarean – repeat, with vacuum and trial of labor attempted  03 = Vaginal – spontaneous  04 = Vaginal – spontaneous, after previous Cesarean  05 = Vaginal – forceps  15 = Vaginal – forceps, after previous Cesarean  06 = Vaginal – vacuum  16 = Vaginal – vacuum, after previous Cesarean |
| Maternal age | Birth records | Maternal age, or if missing Date of delivery- maternal date of birth (in years) |
| Maternal education | Birth records | Did not attend, 1^st^-11^th^ grade, 12^th^ grade no diploma, HS graduate, GED, Some college, Associate, Bachelor, Masters, Doctorate, Professional |
| Body mass index | Birth records | Calculated from height and pre-pregnancy weight |
| Expected payer for delivery | Birth records | Medi-Cal, Tri-Care, Other government programs, Private insurance, Self-pay, Other, Unknown/Unreported |
| WIC participation | Birth records | Yes, No, Unknown |
| Rurality | Birth records | Based on maternal county of residence: https://www.cdc.gov/nchs/data-analysis-tools/urban-rural.html?CDC_AAref_Val=https://www.cdc.gov/nchs/data_access/urban_rural.htm |
| Adequacy of prenatal care | Birth records | Using gestational age at birth, month of prenatal care initiation, and number of prenatal care visits: Kotelchuck Index (PMID: 8092364) |
| Gestational age at delivery | Birth records | Best obstetric estimate |
| Induction of Labor | HCAI, Birth records | ICD9 code 669.7, 763.4 (infant), procedure code 73.0, 73.1, 73.4, 74 or ICD10 code O82, P03.4 (infant), procedure code 10907ZC, 3E033VJ, 3E0P7GC, 10D00Z0, 10D00Z1, 10D00Z2 |
| Body mass index | Birth records | Calculated from height and pre-pregnancy weight |
| Primary attendant for birth | Birth records | Medical Doctor, Doctor of Osteopathy, Certified Nurse Midwife, Licensed Midwife/Registered Nurse Midwife/student nurse midwife/midwife (without license), Other/Other midwife, Unknown or unattended |
| Birthweight for gestational age | Birth records | Calculated using birthweight, gestational age, and sex (PMID: 24777216) |
| Pregestational diabetes | HCAI, birth records | ICD9: 648.0, 250. ICD10: E10, E11, E12, E13, E14, O24.0, O24.1, O24.2, O24.3, O24.9; coded if present in either |
| Gestational diabetes | Birth records | ICD9 code 648.8 ICD10 O24.4, P70.0 (infant) |
| Pregestational hypertension | HCAI, birth records | ICD9: 648.0, 250. ICD10: E10, E11, E12, E13, E14, O24.0, O24.1, O24.2, O24.3, O24.9; coded if present in either |
| Gestational hypertension | Birth records | ICD9 code 642, ICD10 O1 |
| Preeclampsia | Birth records | ICD9 code 642.4, 642.5, 642.6 and/or 642.7, or ICD10 O11, O14.0, O14.1, O14.2, O14.9, O15 |

*HCAI: Health Care Access and Information

**Supplemental Figure 1**. Example of a Candidate Directed Acyclic Graph


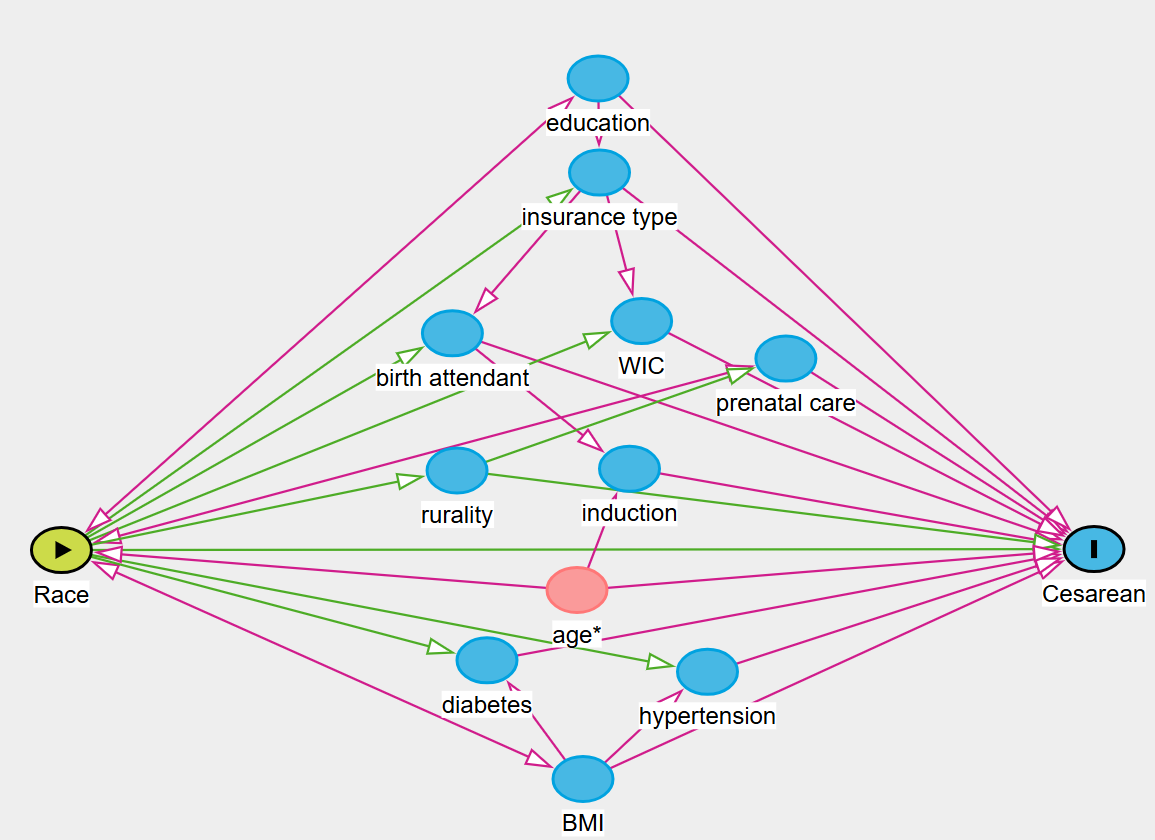


* While by definition, age is not a confounder, we adjusted for it due to its well-documented association with CD and its imbalance across racial groups
